# Molecular survey of the head louse *Pediculus humanus capitis* in Thailand and its potential role for transmitting *Acinetobacter* spp.

**DOI:** 10.1186/s13071-015-0742-4

**Published:** 2015-02-26

**Authors:** Sakone Sunantaraporn, Vivornpun Sanprasert, Theerakamol Pengsakul, Atchara Phumee, Rungfar Boonserm, Apiwat Tawatsin, Usavadee Thavara, Padet Siriyasatien

**Affiliations:** Medical Science Program, Faculty of Medicine, Chulalongkorn University, Bangkok, Thailand; Department of Parasitology, Faculty of Medicine, Chulalongkorn University, Bangkok, Thailand; Faculty of Medical Technology, Prince of Songkla University, Songkhla, Thailand; National Institute of Health, Department of Medical Sciences, Ministry of Public Health, Nonthaburi, Thailand; Excellence Center for Emerging Infectious Diseases, King Chulalongkorn Memorial Hospital, Thai Red Cross Society, Bangkok, Thailand

**Keywords:** *Pediculus humanus capitis*, *Acinetobacter* spp., *COI* gene, *gltA* gene, *rpoB* gene

## Abstract

**Background:**

Head louse infestation, which is caused by *Pediculus humanus capitis,* occurs throughout the world. With the advent of molecular techniques, head lice have been classified into three clades. Recent reports have demonstrated that pathogenic organisms could be found in head lice. Head lice and their pathogenic bacteria in Thailand have never been investigated. In this study, we determined the genetic diversity of head lice collected from various areas of Thailand and demonstrated the presence of *Acinetobacter* spp. in head lice.

**Methods:**

Total DNA was extracted from 275 head louse samples that were collected from several geographic regions of Thailand. PCR was used to amplify the head louse *COI* gene and for detection of *Bartonella* spp. and *Acinetobacter* spp. The amplified PCR amplicons were cloned and sequenced. The DNA sequences were analyzed via the neighbor-joining method using Kimura’s 2-parameter model.

**Results:**

The phylogenetic tree based on the *COI* gene revealed that head lice in Thailand are clearly classified into two clades (A and C). *Bartonella* spp. was not detected in all the samples, whereas *Acinetobacter* spp. was detected in 10 samples (3.62%), which consisted of *A. baumannii* (1.45%), *A. radioresistens* (1.45%), and *A. schindleri* (0.72%). The relationship of *Acinetobacter* spp. and the head lice clades showed that *Acinetobacter* spp. was found in clade A and C.

**Conclusions:**

Head lice in Thailand are classified into clade A and B based on the *COI* gene sequences. Pathogenic *Acinetobacter* spp. was detected in both clades. The data obtained from the study might assist in the development of effective strategies for head lice control in the future. Detection of pathogenic bacteria in head lice could raise awareness of head lice as a source of nosocomial bacterial infections.

## Background

Head lice are obligatory human hematophagous ectoparasites belonging to the Pediculidae family [1]. Head lice infestations or pediculosis occurs throughout the world and is caused by *Pediculus humanus capitis* [2-4]. Molecular techniques have been used for insect species identification and were applied for the biological, evolutionary, phylogenic, and ecological studies. Mitochondrial genes such as cytochrome oxidase subunit I (*COI*) and cytochrome b (*Cyt b*) are typically used for insect species identification studies because of the high inter-species variability and low intra-species variation [5]. Previous studies based on the *COI* and *Cyt b* genes demonstrated that body lice (*P. humanus corporis*) and head lice are separated into three clades, A, B, and C [6]. Head lice could be found in a relatively specific geographic distribution for each clade [7]. Clade A has worldwide distribution [8], clade B is found in Europe, Australia, North America, and Central America [9], and clade C is found in Nepal [10], Ethiopia [11], and Senegal [12].

Head louse transmission occurs by means of clothing, such as, hats, jackets, and scarves, as well as the shared use of hairbrushes and combs [13]. Several reports have suggested that head lice or body lice infestations are vectors of human diseases [14], including epidemic typhus, relapsing fever, and trench fever, via infection with the gram-negative bacteria, *Rickettsia prowazekii* [15]*, Borrelia recurrentis* [16], *Bartonella quintana* [17], respectively. Peleg et al. [18] demonstrated *Acinetobacter baumannii* infections in body and head lice, and the bacteria could cause nosocomial infections and community acquired infections such as pneumonia, bacteremia, endocarditis, and meningitis.

Body lice, instead of head lice, are commonly claimed to be vectors for louse-borne disease transmission because they are associated with high incidences of diseases and high mortality rates, particularly epidemic relapsing fever and typhus, which could be fatal in up to 40% of patients [19]. A recent report demonstrated the detection of body louse-borne pathogens in head lice [14]. Some studies using molecular detection reported that *B. quintana* DNA could be found in head lice collected from homeless individuals in San Francisco, CA, USA [20] and Nepalese slum children [21]. Bouvresse et al. [22] demonstrated *A. baumannii* in head lice collected from elementary school children in Paris, and Kempf et al. [23] showed that *A. baumannii* could be detected in body and head lice collected from healthy individuals from Ethiopia.

In Thailand, data on the genetic identification of head lice and pathogenic bacteria in head lice have not been evaluated. Here, we report the first study of the genetic variations of head lice collected from several geographic regions of Thailand as well as the potential of lice as vectors for *Acinetobacter* spp.

## Methods

### Head louse collections

**Figure 1:**
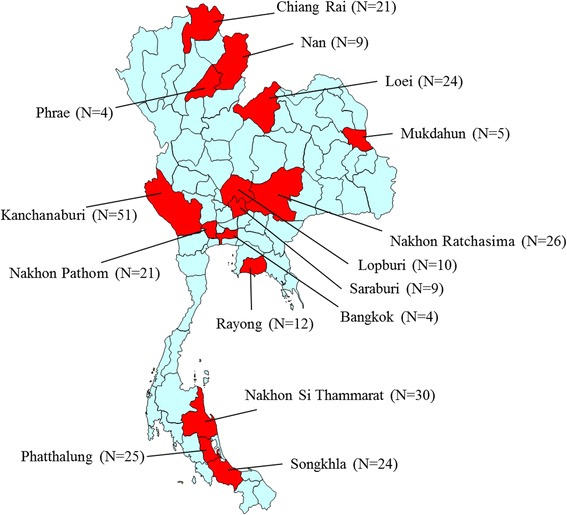
**Map of head lice collection in Thailand.** Red shows the locations of the collection of head lice specimens.

The study was approved by the Institutional Review Board of the Faculty of Medicine, Chulalongkorn University in Bangkok, Thailand (COA no. 424/2014). A total of 275 female adult head lice samples were collected from 26 primary school pupils between 2013 and 2014. The collections were conducted in different geographical regions of Thailand, including the northeastern (Nakhon Ratchasima, Mukdahun, and Loei), northern (Nan, Phrae, and Chiang Rai), central (Bangkok, Nakhon Pathom, Saraburi, and Lopburi), southern (Nakhon Si Thammarat, Songkhla, and Phatthalung), western (Kanchanaburi) and eastern (Rayong) (Figure 1) regions. The head lice were removed from hair using a fine-tooth comb, and all samples were preserved in 70% ethanol before being sent to the Entomology Laboratory, Department of Parasitology, Faculty of Medicine, Chulalongkorn University and stored at −20°C until use.

### DNA extraction

The head lice specimens were removed from the 70% ethanol by being washed three times with phosphate buffer saline (1XPBS), and then an individual head louse of each sample was homogenized in 200 μl of lysis buffer G and 20 μl of proteinase K. The genomic DNA was extracted using a DNA extraction kit, Invisorb® spin tissue mini kit (STRATEC molecular GmbH, Berlin, Germany) following the manufacturer’s instructions. The extracted head lice DNA was eluted in 40 μl of elution buffer, and the concentration was measured using Nano drop 2000c (Thermo-scientific, USA). The genomic DNA was stored for an extended time at −20°C until the next stage of the investigation.

### PCR amplification

Degenerate oligonucleotide primers were designed based on the *COI* sequences of the head lice (*P. humanus capitis*) and *Phthirus pubis* obtained from the GenBank database (GenBank:EU493419, EU493427, EU493433, EU493435, EU493437, EU493439, EU493441 for *P. humanus capitis* and EF152554, AY696000 to AY696005 for *Ph. pubis*) as forward primer 5'-GGTACTGGCTGGACTRTTTATCC-3', and the degenerate reverse primer sequences were 5'-CTAAARACTTTYACTCCCGTTGG-3'. The primers were synthesized by 1st BASE Oligonucleotide (Oligo) Synthesis services company (1st BASE Laboratories, Malaysia). PCR was used to detect *Bartonella* spp. or *Acinetobacter* spp. The DNA in the head louse samples were targeted from the *gltA* gene and *rpoB* gene for *Bartonella* spp. [24] and *Acinetobacter* spp. [23], respectively. The PCR reaction was set up in a final volume of 25 μl containing approximately 50 ng/μl of extracted DNA, 10 μM of each primer, 10X*Taq* buffer, 2.5 mM of dNTPs, 2.5 mM of MgCl_2_ and 1 unit of *Taq* DNA polymerase (Fermentas, Pittsburgh, PA); double distilled water was the negative control. The PCR amplification conditions were as follows: initial denaturation at 95°C for 3 minutes; 40 cycles of 95°C for 1 minute, 50, 60, and 62°C for *COI*, *gltA*, and *rpoB* gene, respectively for 1 minute and 72°C for 1 minute; and the final extension at 72°C for 7 minutes. The PCR amplicons were determined via 1.5% agarose gel electrophoresis, stained with ethidium bromide, and visualized with Quantity One Quantification Analysis Software version 4.5.2 (Gel DocEQ System; Bio-Rad, Hercules, CA).

### DNA cloning and sequencing

The PCR amplicons were ligated into pGEM-T Easy Vector (Promega, Madison, WI) using T4 DNA ligase. The recombinant plasmids were transformed into competent cells (*Escherichia coli* DH5α strain), and then the recombinant plasmids were screened using the blue-white colonies system. The colonies suspected to contain the insert gene were cultured, and the plasmid DNA was extracted using the Invisorb® Spin Plasmid Mini kit (STRATEC molecular GmbH, Berlin, Germany) according to the manufacturer’s instructions. The purified plasmids were sequenced by 1st Base Laboratories, Malaysia.

### Sequence analysis and phylogenetic tree construction

The obtained nucleotide sequences were analyzed by comparison with the nucleotide sequence in the GenBank database using BLASTN (http://blast.ncbi.nlm.nih.gov/blast/Blast.cgi), and all the nucleotide sequences from this study were submitted to the GenBank database. The nucleotide sequences of each region were aligned, and the percentage of the intra-specific variation was calculated using BioEdit Sequence Alignment Editor, Version 7.1.9. A phylogenetic tree was constructed via the neighbor-joining method using Kimura’s 2 -parameter model implemented in MEGA6.06, and the tree was tested using 1000 bootstrap replicates and compared with the reference sequence (clade A, B and C). *Pediculus schaeffi* accession no. AY695999 was an outgroup.

## Results

### Sequencing and phylogenetic tree analysis

**Figure 2:**
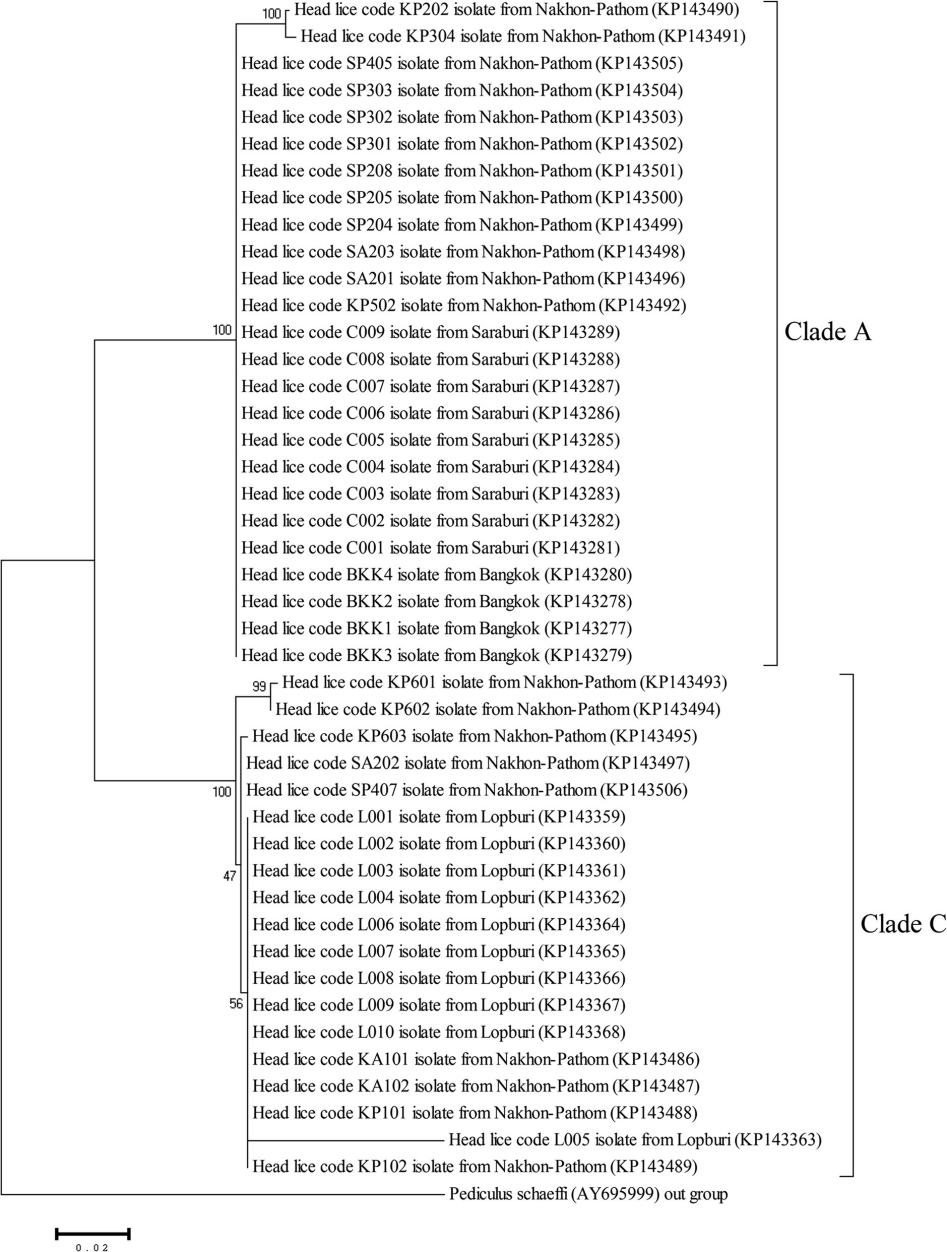
**Phylogenetic tree of head lice constructed from partial** ***COI*** **sequences from the central region of Thailand.** The phylogenetic tree using the neighbor-joining method with the Kimura 2 parameter model with the bootstrap values of 1000 replicates. *P. schaeffi* accession number AY695999 was used as an outgroup.

In total, 275 adult head lice were collected from 6 geographical regions of Thailand, and the partial *COI* gene was amplified from all head louse samples. Most of the sequences contained 599 bp, and 4 samples contained 603 bp of the nucleotide sequences (Genbank:KP143370, KP114375, KP143490, and KP143491); all these sequences have insert GGAG nucleotides with 98% identity with sequences in the GenBank database (GenBank:KC685849). The comparisons of all sequences with the GenBank database were similar to the comparisons of *P. humanus capitis*. The entire sequence was assigned GenBank numbers (GenBank:KP143232-KP143506). The intraspecific variation analysis found that head lice from the northern region showed variation within the species more than the variation found in head lice from the other regions (0-17%) (data not shown). The phylogenetic tree based on the *COI* gene revealed that the head lice were clearly classified in clade A and clade C (Figure 2).

### Detection of *Bartonella* spp. and *Acinetobacter* spp.

**Figure 3:**
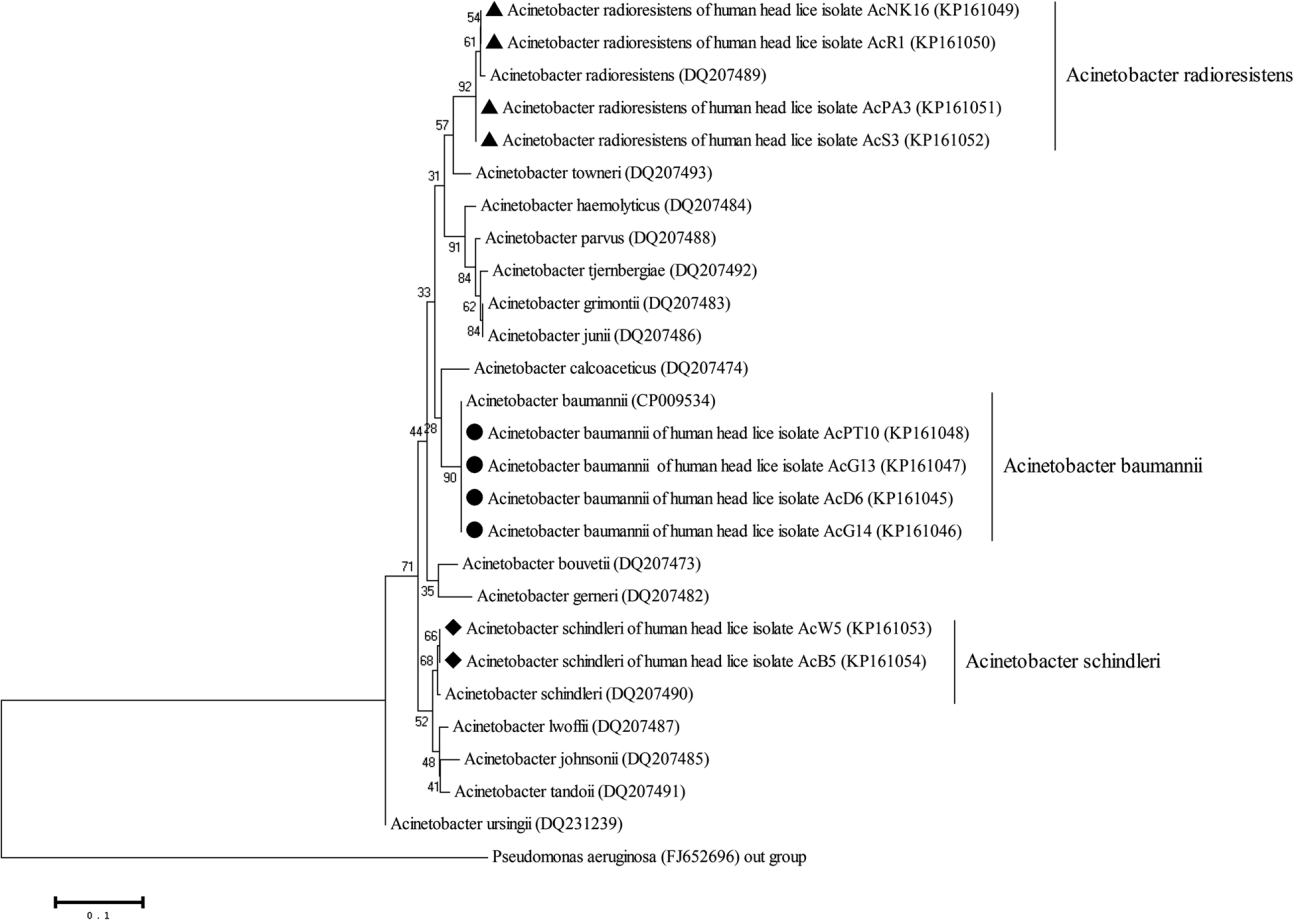
**Neighbor-joining trees were constructed of the** ***rpoB*** **gene of** ***Acinetobacter*** **spp. in head lice DNA.** The phylogenetic tree relationship of *Acinetobacter* spp. detected in head lice was compared with the reference sequences strain using the Kimura 2 parameter model with the neighbor-joining method by testing with 1000 bootstrap values, and *Pseudomonas aeruginosa* accession number FJ652696 was used as an outgroup.

In this study, the PCR investigation of *Bartonella* spp. and *Acinetobacter* spp. in the DNA of head lice did not detect *Bartonella* spp. DNA; the DNA of *Acinetobacter* spp. was detected in 10 samples (3.62%) of head lice DNA (Genbank:KP161045-KP161054). The nucleotide sequences of *Acinetobacter* spp. were compared with the GenBank database sequences and were identified as *A. baumannii*, *A. radioresistens*, and *A. schindleri* with 98-100% identity. The infection rate of *A. baumannii*, *A. radioresistens*, and *A. schindleri* in head lice in this study was 1.45%, 1.45%, and 0.72%, respectively (Table 1). The phylogenetic tree demonstrated that 3 species of *Acinetobacter* spp. were classified in the same group as the reference sequence strain (Figure 3). The DNA of *Acinetobacter* spp. was found in clade A and clade C of the head lice (Table 1). *Bartonella* spp. was not detected in the head lice in this study.

**Table 1:** **Head lice in clade A and C and** ***Acinetobacter*** **spp. detection in Thailand**

**Regions**	**Sample no. (n)**	***COI*** **gene**		***rpoB*** **gene**		**Species identification**
		**Clade A (n)**	**Clade C (n)**	***Acinetobacter*** **species (n)**	**% infection rate in Thailand**	
Northern	34	27	7	3	1.09	*A. baumannii* (n = 1)
						*A. radioresistens* (n = 2)
Central	44	25	19	0	0.00	
Northeastern	55	37	18	2	0.72	*A. baumannii* (n = 2)
Southern	79	46	33	3	1.09	*A. baumannii* (n = 1)
						*A. radioresistens* (n = 1)
						*A. schindleri* (n = 1)
Western	51	47	4	1	0.36	*A. schindleri* (n = 1)
Eastern	12	11	1	1	0.36	*A. radioresistens* (n = 1)
Total	275	193	82	10	3.62

## Discussion

Head louse infestation remains a health problem of children in Thailand. Using mitochondrial *COI* gene sequences, we successfully demonstrated genetic variations in head lice collected from different geographical regions of Thailand. The phylogenetic tree analyses on the head louse sequences classified the lice into 2 clades. In accordance with previous reports, Kittler et al. and Reed et al. revealed that the phylogenetic tree analyses of their studies examined the *COI* sequence data, which showed 3 clades of human lice collected from Europe, Africa, and Asia. One clade contained head and body lice (clade A), whereas clade B and clade C each contained only head lice [9,10,25]. This study demonstrated that head lice in Thailand belong to clade A and C. Both clades could be found in all regions of the country. The data confirm that clade A has worldwide distribution [8]. Previous studies reported that clade C is found in Nepal [10], Ethiopia [11], and Senegal [12]; this is the first report of clade C found in Thailand.

To determine whether head lice could transmit pathogenic bacteria, the primer sets targeting the *rpoB* gene and *gltA* gene of *Acinetobacter* spp. [23] and *Bartonella* spp. [24] were used to detect bacterial DNA in the head louse samples. Three *Acinetobacter* species (*A. baumannii*, *A. radioresistens* and *A. schindleri*) were detected in 10 samples; 4 samples were positive for *A. baumannii*, *A. radioresistens,* and 2 samples were positive for *A. schindleri.* Previous reports demonstrated that *A. baumannii* is the most commonly found species in head and body lice in Ethiopia, Portugal, the Netherlands, and France [23,26]. The DNA of *A. baumannii* was more frequently isolated from body lice than from head lice [19]. Punpanich et al. [27] reported that children at the Queen Sirikit National Institute of Child Health (QSNICH), Thailand were found to have a nosocomial infection caused by *A. baumannii*.

*B. quintana* is a facultative intracellular bacterium that causes diseases including trench fever, chronic bacteremia, endocarditis, bacillary angiomatosis and chronic lymphadenopathy [28]. The bacterial DNA of *B. quintana* was detected in head lice collected from homeless individuals from USA [20] and Nepalese slum children [21]; however, *B. quintana* DNA was not detected in this study. Our result is similar to that of a previous report conducted on head lice of elementary school children in Paris; the study was unable to detect *B. quintana,* whereas it found *A. baumannii* [22].

The data obtained from this study might be used to develop effective planning for head louse control. The detection of pathogenic bacteria in head lice is useful for monitoring the possible head louse-borne pathogens in humans.

## Conclusions

This report is the first study using molecular techniques to investigate head lice in Thailand and the first to describe *Acinetobacter* spp. in human head lice collected from school children in Thailand. The techniques could be used for classification and determination of the genetic variations of head lice, and the report provides fundamental data for further epidemiological studies of head lice in Thailand. Detection of pathogenic bacteria in head lice is crucial for monitoring the head louse-borne pathogens transmitted to humans. Future studies that include epidemiological data, more geographical areas, and a larger sample size of head lice are required; future studies might provide insights into the evolution of bacteria and vector hosts as well as into whether head lice play a role in spreading *Acinetobacter* spp. or other pathogens to human hosts.
